# Review of Portable and Low-Cost Sensors for the Ambient Air Monitoring of Benzene and Other Volatile Organic Compounds

**DOI:** 10.3390/s17071520

**Published:** 2017-06-28

**Authors:** Laurent Spinelle, Michel Gerboles, Gertjan Kok, Stefan Persijn, Tilman Sauerwald

**Affiliations:** 1 European Commission—Joint Research Centre, 21027 Ispra, Italy; laurent.spinelle@ec.europa.eu; 2 VSL Dutch Metrology Institute, 2629 JA Delft, The Netherlands; GKok@vsl.nl (G.K.); SPersijn@vsl.nl (S.P.); 3 Laboratory for Measurement Technology, Universitaet des Saarlandes, 66123 Saarbruecken, Germany; t.sauerwald@lmt.uni-saarland.de

**Keywords:** PID based sensors, semiconductor and amperometric sensor, mini GC, portable on-line measuring devices, benzene, volatile organic compounds

## Abstract

This article presents a literature review of sensors for the monitoring of benzene in ambient air and other volatile organic compounds. Combined with information provided by stakeholders, manufacturers and literature, the review considers commercially available sensors, including PID-based sensors, semiconductor (resistive gas sensors) and portable on-line measuring devices as for example sensor arrays. The bibliographic collection includes the following topics: sensor description, field of application at fixed sites, indoor and ambient air monitoring, range of concentration levels and limit of detection in air, model descriptions of the phenomena involved in the sensor detection process, gaseous interference selectivity of sensors in complex VOC matrix, validation data in lab experiments and under field conditions.

## 1. Introduction

Volatile organic compounds (VOCs) are hazardous compounds that may cause damage to humans with chronic exposure [1]. The main compounds of interest consists of aromatics such as benzene, toluene, xylene, and ethylbenzene (BTEX) and aldehydes, such as formaldehyde and acetaldehyde. The World Health Organisation (WHO) Air Quality Guideline for Europe [2] establishes guidelines values for toluene, 260 μg/m^3^ over 1 week, for formaldehyde, 100 μg/m^3^ over 30 min and for tetrachloroethylene, 250 μg/m^3^ over 1 year. Additionally, it sets the guideline values for benzene corresponding to the concentrations levels associated with an excess lifetime risk of 1/10,000, 1/100,000 and 1/1,000,000 equal to 17, 1.7 and 0.17 μg/m^3^, respectively. In 2010, the WHO Guidelines for Air Quality in Indoor Air confirmed the guideline values given in 2000 for benzene [2].

In Europe, the European Air Quality Directive (AQD [3]) defines as mandatory the monitoring of benzene in ambient air. The AQD states that the reference method for the measurement of BTEX consists of active or on-line sampling followed by desorption and gas chromatography [4]. This method is time consuming, expensive to implement and it needs skilled personnel to perform complex operations. While some implementation of this method may be transportable, it is not easily portable prohibiting estimation of the population exposure. Therefore, the reference method is not for dense networks of BTEX automatic monitoring sites covering large areas or for the estimation of real-time human exposure. However, where the upper assessment threshold (UAT) is not exceeded the AQD allows the use of indicative measurements without restriction in the zones while their implementation permits a reduction of 50% of the minimum reference measurements where the UAT is exceeded. The Directive does not specify any indicative methods but it requires the selected indicative method to meet a defined Data Quality Objective (DQO). The AQD set the following requirements regarding benzene measurements:

- the DQO is 30% for indicative measurements. It is defined as the relative expanded uncertainty of measurements and it shall be assessed in the region of the limit value (LV);
- the LV for the benzene annual mean is 5 μg/m^3^ (about 1.5 ppb at 20 °C and 101.3 kPa). Other important values defined in the AQD consist of the Upper Assessment and Lower Assessment Thresholds (UAT and LAT) which correspond to 3.5 and 2 μg/m^3^, respectively.

In the future, air quality assessment should rely on exposure based monitoring of air pollutants with higher spatial resolution [5] even though using measurement methods with lower data quality (the so-called indicative measurements in the AQD). The need for mobile applications and better spatial coverage can only be satisfied by reducing size and costs of monitoring devices using for example low-cost and portable sensors. Commercial low-cost sensors represent a big opportunity for developing networks of VOC measurements able to monitor large areas with higher spatial resolution at a lower cost than the reference measurements method [2].

In comparison to other indicative methods such as diffusive samplers [6], micro-sensors are able to supply near to real time air pollution measurements by electronic means. This make it possible to assess the effect of short term action plans (AQD, art. 24) and simplify reporting of air quality to the Internet (AQD, art. 26 and article 23 of the INSPIRE Directive [7]).

The existing technology of gas sensors has allowed the introduction on the market of various types of low-cost sensors for air pollution monitoring such as metal oxide sensors (MOx), amperometric or potentiometric electrochemical cells, photo-ionisation detectors (PID), portable and micro GC (μGC). However, few or no performance evaluation studies exist to demonstrate that the DQO of the AQD can be reached. There have been only a few successful previous research studies with custom sensors and/or commercial sensors for quantitative determination [8,9,10] or identification [11] of low level VOC and inorganic gases. Moreover, in general the information regarding the sensor metrological specifications and performances is varied and difficult to compare.

In 2005, Yamazoe [12] claimed that benzene levels in ambient air below 100 ppb were far beyond the capabilities of commercially available gas sensors. In about a decade the technological progress resulted in an improvement of sensor sensitivity and a few systems are able now to reach the ppb or in a few cases sub-ppb level of sensitivity for monitoring benzene in ambient air.

Hereafter, the performances of commercial VOC sensors for the monitoring of air pollution at ppb levels are compared. The original goal was to compile a list of cheap sensors, hoping to find items within an initial price limit of 1000 €. This limit had to be increased up to 5000 € because of the lack of commercial instruments within the 1000 € range. A summary of the advantages and disadvantages of each sensor technology is given focusing on benzene sensors. However, where easily reported, information on BTEX and other aromatics or VOCs is also given. The majority of the information compiled is drawn from the sensor datasheets provided by the manufacturers. It was generally not possible to confirm the sensor performances claimed by the manufacturers since independent evaluations are not widely available. The authors tried to tabulate comparable characteristics for all sensors reported though they cannot take responsibility for the accuracy of the information given in the datasheet of the equipment.

## 2. Principle of Operation and Type of Sensors

Nearly all small commercial VOC sensors are based on one of the six principles of operations that are presented in the following subsections:

- Photo-ionization detectors (PID), both portable hand held instrument and Original Equipment Manufacturers (OEMs),
- OEM electrochemical sensors either of amperometric or potentiometric type,
- OEM metal oxide sensors (MOx) with change of conductivity instead of chemical reaction,
- Optical sensors including UV portable spectrometers,
- Portable or micro-gas chromatograph (μGC) that combines micro column with MOx or PID OEM as detectors. Flame ionization detectors (FID) are generally not considered in this review because of the need of an external hydrogen source for operation. Bench top instruments are excluded in this category for the lack of handiness and their high price range,
- and electronic noses and sensor-arrays.

Only a few literature reviews of low-cost sensors for monitoring benzene in ambient air in the ppb range exist. Most of the publication cover the sensors for VOCs measurements. Among the initial ones, Ho et al. [13], in 2001, presented a review of the sensors that were capable of detecting and monitoring VOC for long-term air pollution monitoring. The review included chromatographic/spectrometric sensors; conductimetric MOx sensors, amperometric/potentiometric sensors; acoustic wave sensors; and optical sensors. The authors found that only portable gas chromatographs, ion mobility spectrometers (IMS) and portable mass spectrometers could reach a limit of detection in the ppb range.

However, these instruments are too expensive and cannot be classified as low-cost sensors. The authors concluded that the most viable sensors for in-situ chemical sensing were likely to be polymer absorption chemiresistors, MOx sensors, fiber-optic sensors, and surface-acoustic-wave sensors. This conclusion was drawn in absence of information on the sensitivity, selectivity and any type of evaluation study. It was based on the simplicity and robustness of these types of sensors.

A recent review published by Szulczynski et al. [14] depicts the current state of commercially available sensors for the detection of VOCs in both outdoor and indoor air over a high range of concentrations. In particular they describe the main technologies (electrochemical, nondispersive infrared, pellistor and photoionization) and give a list other types supported by numerous references. They finally gather some metrological parameters (range, accuracy, resolution, LOD, sensitivity and response time) based on the information available from the manufacturer. The authors concluded that the current sensors technologies present advantages, in particular economic. However, the sensors face limitations, such as too high limit of detection or poor selectivity.

In the following sections, for each of the six types of sensors previously cited, the principle of operation is described, the performance and availability of commercially sensors is listed, and the main results and improvements from research papers are given.

## 3. Photo Ionization Detector

### 3.1. Principle

Photo-ionization detectors (PIDs) use high-energy photons, typically in the ultraviolet (UV) range. The use of UV light to excite the molecules results in the ionization of gas molecules. The energy of the photons is typically in the range of 10 eV, so that only gases with low ionization energy e.g., organic vapour, can be ionized. The main air constituents, oxygen and nitrogen, do not form ions at this photon energy. The resulting ions produce an electric current proportional to the signal output of the detector. More molecules are presents in the air, more ions are produced, and higher will be the resulting current. Finally, the ions recombine after the detector to reform the original molecules. Intrinsically, PIDs sensors not selective as they ionize everything with an ionization energy less than or equal to the lamp output.

### 3.2. Commercially Available Sensors

The main OEM PID sensors include the Ion Science (Cambridge, UK) model ppb MiniPiD white, AlphaSense (Great Notley, UK) model PID-AH for VOCs, Mocon-Baseline (Lyons, CO, USA) models piD-TECH eVx Blue 045-014 and piD-TECH plus 043-235. These small sensors can reach a low limit of detection (LoD) due to their high sensitivity to benzene up to sub-ppb for the MiniPiD white, PID-AH and piD-TECH eVx Blue, respectively (see Table 1 and Appendix A).

Unfortunately, none of these sensors are selective to a particular VOC, and even with a xenon lamp of 9.6 eV benzene, toluene and xylene cannot be distinguished as the ionisation potential of these compounds is lower than the energy of the lamp [27].

A few manufacturers propose portable instruments displaying real time measurements: Dräger (Houston, TX, USA) model Multi-PID 2, Mocon-Baseline (Lyons, CO, USA) model VOC-Traq, GrayWolf (Shelton, CT, USA) models Advanced Sense and Direct Sense, Ion Science (Cambridge, UK) model Club Personal and Tiger Select, RAE Systems (San Jose, CA, USA) models UltraRAE 3000 and model ppbRAE 3000, PID Analysers (Sandwich, MA, USA) model 102+. Some of them also include a selective absorbing cartridge for benzene (Dräger model Multi-PID 2, Ion Science model Tiger Select and RAE Systems model UltraRAE 3000). Unfortunately, the sensitivity of the portable instruments is lower than the OEM sensors, especially for the ones selective to benzene. This is likely because of the added absorption cartridge. The best limit of detection for benzene was found to be 50 ppb for the Dräger and RAE instruments and 10 ppb for the Ion Science Tiger Select. Generally, the price of OEM PIDs is around 500 € while the price of portable hand held PID sensors is about 5000 €.

### 3.3. Literature Survey

Peng et al. [28] proposed a PID sensor signal generation system improvement. Compared with commercial instruments the sample was directed to flow across the lamp window, rather than toward the lamp. Moreover, a new and simple automatic self-cleaning technique had also been adopted, which eliminates contaminants more effectively and more substantially, reduces the drift. The use of new electrodes had effectively reduced the background noise and dead volume of the PID. As a result of this new design the PID should be more compatible with rapid portable GC in environmental monitoring, because of the elimination of most of the previously necessary tedious cleaning and calibration.

## 4. Amperometric Sensors

### 4.1. Principle of Operation

Electrochemical gas sensors are one of the oldest known technologies and widely used for concentration measurements. There are different basic principles for electrochemical sensors: potentiometric sensors if a difference of potentials is measured or amperometric if the current of a redox reaction is measured. The electrochemical reaction is due to the transfer of a charge from the electrode to the electrolyte. This electrolyte can be a solid [29], gel-like (or an organic gel as in the case of City Technology), liquid or gaseous electrolyte (e.g., for SGX Sensortech, Corcelles-Cormondreche, Switzerland). This process is based on the chemical reaction at the electrode and the transport of charges throughout the electrolyte. Electrochemical cells require at least two electrodes. Nowadays, the majority of amperometric sensors for measurement at low levels includes three electrodes (measuring/working, counter and reference electrodes). Some manufacturers also add a fourth electrode for monitoring physical changes and drift in the sensor architecture (e.g., for AlphaSense and Membrapor, Wallisellen, Switzerland).

An amperometric sensor is made of a measuring and a counter electrodes together with an additional reference electrode. The gaseous species to be measured diffuses trough the sensor’s membranes and to the measuring electrode. A direct electron transfer takes place which produce an internal current which gives a measured electric current proportional to the gas concentrations [30] following the Nernst Law for electrochemical reactions [31].

These are low cost, low power, compact sensors and in general, their response time is about 120 s depending on the air temperature. As with PIDs, electrochemical cells are broadband sensors, but with a different profile: PIDs show a better sensitivity than electrochemical cells for VOCs. If one wishes to measure a VOC with electrochemical cells, then it is necessary to optimize the electrochemical sensor to the target VOC. In fact, amperometric sensors show little selectivity and a limit of detection down to the high ppb range. This type of sensors can be tuned to a specific target gas in many ways. One of the most logical way is to target a specific chemical reaction of the electrochemical reaction controlling the selectivity. Some physical characteristics of the diffusion barrier (e.g., porosity or pore distribution [32]) can be adjusted for specific molecules, in addition to the bias voltage between the reference and the counter electrode, the type of electrolyte, the material of the measuring electrode etc. which impact the selectivity such types of gas sensor.

Usually, the size of these sensors is about 20 mm and they have a very low electrical consumption owing to the low electrical current signal generated. The main power requirement is the amplification of the very low-level signal required to read the measurement. The electrodes composition also gives selectivity and sensitivity to diffuse target gases such as VOCs [33], NH_3_ [34], O_3_ [35], NO_2_ [36] and NO [37,38]. The electrodes can be composed of different materials and different supports. For example, the SGX Sensortech uses porous PTFE with catalytic materials. The most used electrolytic sensors have a very high range of detection [38] but they can nowadays achieve low detection limits [39]. The usual measuring range for VOCs is between 100 ppb and 20 ppm. For all the manufacturers, the deviation from linearity tends to be similar in all the sensors with about 2–5% of error, up to 10% in some cases. By selecting different electrode materials, the reaction rate to several gases can be fine-tuned, but not completely eliminated.

Most of the amperometric sensors need humidity to function properly. In fact, certain electrolytes can be damaged by very low humidity, leading to a bias in the measurements. Solid-state material-based sensors are not so dependent on ambient humidity. The temperature also has an influence on the sensor response, but this interference can be modelled and compensated. Wind velocity, in particular in ambient air applications, can also have an influence on the chemical equilibrium on the [40] or the diffusion through the membrane of these sensors. The sensors show long-term stability with drift values between 2% and 15% per year, for example, for the Nemoto and SGX Sensortech devices. The calibration of these sensors follows the Nernst law and they can be well calibrated for the gases to be measured using either a linear or a logarithmic [33] function. The evaluation of measurement uncertainty for amperometric sensors is described in Helm et al. [41].

Another type of electrochemical sensor should be distinguished as they use an ionic liquid as electrolyte. Gębicki and Kloskowski describe in their paper [42] an ionic liquid-based sensor prototype able to detect benzaldehyde in air in the ppm range. The use of ionic liquid gives them the capacity to tune different physical and electrical parameters such as viscosity, conductivity or water solubility. They then study the impact of these parameters on the sensor performance.

### 4.2. Commercial Sensors

No commercial manufacturer of potentiometric sensors for VOCs measurement could be found, while there are a few well-known manufacturers of amperometric sensors for VOCs. They generally propose 3-electrodes amperometric sensors adjusted to measure ethylene oxide with a number of inorganic and organic interfering compounds (see Table 2 and Appendix A):

- City Technology (city, UK) model 3ETO CiTiceL and 4ETO CiTiceL, 7ETO CiTiceL,
- Alphasense LTD (UK) model ETO-A1, ETO-B1,
- Membrapor AG (city, CH) model ETO/M-10 and ETO/C-20,
- and SGX Sensortech (CH) model EC4-10-ETO.

The detection limit for all these sensors (for ethylene oxide) is too high for air quality monitoring with the best figure reaching 50 ppb. All sensors exhibit similar sensitivity, between 1.9 and 2.8 μA/ppm, leading to a low signal when measuring VOCs in the ppb or sub-ppb range that cannot be distinguished from the electronic noise of the sensors or the measuring data acquisition systems even though a high load resistance or amplifying gain is used. The price of OEM amperometric sensors is generally about 100 €.

### 4.3. Literature Survey

A few articles can be found regarding potentiometric sensors which use electrolytes (e.g., sodium super ionic conductor NASICON) [51,52,53,54,55]. These papers provide both mechanisms of reaction and insights for improvement of this technology. However, all these sensors exhibit a limit of detection in the low ppm range or sub-ppm for the best ones. They remain far from our objective of low ppb, high ppt limits of detection.

Knake et al. [56], presented the direct amperometric detection of low levels of formaldehyde in the gas phase using an acidic electrochemical cell based on a gold coated Nafion membrane as working electrode. The sensor was found to show a linear response with a limit of detection of 13 ppb up to a full scale of 10 ppm. It suffered from a face wind effect and humidity. Cross sensitivities to a number of organic and inorganic gases were also observed. The interferences from NO, NO_2_ and SO_2_ was corrected using an aluminium oxide filter on which formaldehyde was selectively adsorbed. By calculating the difference of the measurements with and without a filter, a clear signal for formaldehyde could be obtained in presence of the interfering compounds.

Sekhar and Subramaniyam [57] presented a set of three electrochemical mixed potential gas sensors for the detection of benzene, toluene, ethylbenzene and xylenes. The sensor configuration made from strontium-doped lanthanum chromite (La_0.8_Sr_0.2_CrO_3_, abbreviated as LSCO) electrode and platinum (Pt) electrode with yttria-stabilized zirconia electrolyte exhibited maximum sensitivity and selectivity to BTEX. The authors found a detection limit better than 0.5 ppm using a mixed potential tape cast sensor. The mixed potential was found to vary linearly with BTEX concentration for all the studied sensors. To decrease the detection limits to the ppb levels the authors considered the use of a cold-wall set-up and heterogeneous catalysis studies.

## 5. Resistive Sensors

### 5.1. Principle of Operation

The transducing mechanism of these sensors consists of a metal oxide that changes its electrical properties when exposed to different ambient gases. In commercial sensors, the property overwhelmingly measured in metal oxide sensors is the resistance or conductivity. Tin oxide (SnO_2_) is most used because it has a wide reactivity and strong changes in resistance. The model widely accepted is that tin oxide form grains and the boundary of those grains dominate the conductivity. In presence of an oxidizing gas, normally oxygen in ambient air, the gas molecules react with the tin oxide trapping electrons on the surface, creating a positive charge space that acts as a barrier to conductivity.

Resistive sensors are usually smaller compared to amperometric ones, with a size of a few millimetres and a weight about a few grams. They need high temperature for the reactions to take place at a faster rate so a heater is usually incorporated into the sensor. They respond to a wide range of concentrations of the gases: from a few ppb for gases like NO_2_ [58] to several thousand of ppm for other gases [39]. However, the signal to noise specification provided by the sensor is usually not very clear and none of them have methods to deal with the mixture problems and usually just provide tables of the equivalent gas concentration cross sensitivity to other gases. The gas desorption tends to be very slow, increasing the length of time needed to make a measurement. The times can be as high as 45 min [59] but in most of the cases, their response time is in the range of the few minutes [39]. This kind of sensors are like PID not specific to individual organic compounds. Moreover, these sensors do additionally respond to inorganic reducing and oxidising gases like e.g., CO or NOx. To improve their selectivity, manufacturers typically incorporate different dopants or filters. Temperature and humidity are also important interferents of the signal and have to be controlled or measured with precision so they can be extracted and their influence can be modelled. The worst problem of this type of sensor is probably their stability. In fact, the response changes over time and the sensors needs to be recalibrated more regularly. Manufactures do no provide much information about the drift or stability. 

For a gas sensor at stationary conditions, many authors report an empirical relation for gas concentration and the sensor response:

(1) $$G-G_{0}=a\;P^{b}$$

where *G* is the conductivity, *G*_0_ is the conductivity at zero gas concentration, *P* is the gas concentration and *a* and *b* are constants [60]. This empirical relation can be described by the change of negative surface charge in a chemical surface reaction with the reducing gas modelled as a stationary chemical reaction using the mass action law [61]. The limit of detection is often not studied in detail, especially in literature surveys presenting new sensor materials. To compare results from different literature studies the measurement results have been extrapolated using Equation (1), usually with the assumption of *b* = 1. However, like all extrapolations, these results are only indicative, but there is also the possibility to extract temporal parameters from the response curves, such as integral of the response curve [62] or other methods like extracting parameters by Principal Component Analysis from the curves of resistance after a change of temperature [60]. Other authors use non-parametric methods such as neural networks [39], linear partial least squares (PLS) and spline fitting of the data [60] or even decomposition in functions such as Bessel decomposition [63] and Fourier or wavelet expansions [64].

They are generally compact, low cost and need higher power than PIDs. MOx sensors also respond to inorganic gases, so one should not use them to measure low concentrations of VOCs where gases such as NO, NO_2_ or CO are also present in higher concentrations. Thus, when using MOx sensors, information about long-term stability, cross-sensitivity to gaseous interfering compounds and humidity sensitivity is also important in order to correct sensor response. MOx sensors are advisable when sensing VOCs that are not measured by PIDs (e.g., many CFCs).

A few studies show that the most sensitive oxides for VOCs include the four following types: WO_3_, SnO_2_, In_2_O_2_ and ZnO [65,66,67]. However, the probability than a complex gas mixture contains higher concentrations of interfering gases compared than the target compounds, the typical lack of intrinsic selectivity of these metal oxide gas sensors remains the limiting factor to a possible mass production. Several methods have been studied to improve the selectivity of SnO_2_ gas sensors. For example, we can quote the use of physical or chemical filters [68,69], the doping of the sensitive element [69,70], the combination of several detection systems on the same matrix (electronic noses) [71,72,73] or the operation of sensors under different temperatures [74] or dynamic regimes [75].

### 5.2. Commercially Available Sensors

A lot of MOx sensors for VOCs are commercially available: AMS (S) iAQ-100, iAQ-2000, iAQ-engine and AS-MLV, Unitec (I) SENS 3000 or SENS-IT, UST (D) GGS-1330T, UST-3330T, UST 8330T, SGX SensorTech (CH) MICS-5121, MICS-5521 and MICS-VZ-87, Figaro (J) TGS 2201, 2600, 2602, 8100 and 822, FIS (J) SP3_AQ2 and Synkera Technologies (USA) VOC Sensor (P/n 731). Their characteristics are listed in Table 3 and Appendix A. A modified version of the Aeroqual VM with lower limit of detection is also included although it does not appear to be readily commercially available. As for amperometric sensors, the MOx sensors appear to give a limit of detection that is too high for air quality monitoring with the best value reaching 100 ppb except for the Unitec SENS3000/SENS-IT. However, the tests of this sensor carried out by USA Environmental Protection Agency showed noisy data limiting the level of sensitivity claimed by the manufacturer [76].

Among the Figaro sensors (Figaro Engineering, Osaka, Japan), the majority of sensors are designed for high concentration of gas compounds, generally over 1 ppm. The most suitable Figaro sensors for VOC monitoring consist of the TGS 2600 (for i-butane, ethanol and methane), TGS 2602 (for toluene), TGS 2201 (for *i*-butane and methanol). The TGS 8100 exhibits similar results as TGS 2600 and 2602 while the dimension and power consumption is reduced.

The responses of Figaro sensors are generally presented with a linear plot with logarithmic axis of Rs/R0 versus concentration showing that Equation (1) can be rewritten in a simplified form as in Equations (2) and (3):

(2) $$\log_{10}\frac{R_{s}}{R_{0}}=a+b\;\log_{10}p$$

(3) $$p={\left(\frac{R_{s}}{R_{0}}10^{-a}\right)}^{\frac{1}{b}}\;\mathrm{or}\;p=\sqrt[{b}]{\frac{R_{s}}{R_{0}}10^{-a}}$$

where *R_s_* is the sensor resistance in displayed gases at the measured concentration *p* and *R*_0_ is the sensor resistance in clean air. The advantage of using these equations is that the sensor response has a linear relationship with the analyte while the major drawback is the huge estimation error due to the logarithmic law. 

The ETL2000 measurement device is a sensor platform mounting Sens3000 sensors and manufactured by UNITEC. They are based on a thick-film MOx sensor for benzene which has been evaluated during a seven week-long measurement campaign in an urban background location [77]. Fifteen min averages from the ETL2000 were compared with the measurements from an AirmoBTX 1000 reference analyser. The ETL2000 was able to reproduce the concentration distribution within the higher value range. However, below 3 μg/m^3^, a decrease of sensitivity and selectivity was observed. The ETL2000 may be an alternative for more expensive reference measurement methods in places where the expected benzene concentrations are rather high. The price of MOx sensors is generally about 50 €, with some exceptions for more sophisticated systems.

### 5.3. Literature Survey

In 2002, Mabrook and Hawkind [97] developed a sensor where a sensitive material made of titanium dioxide dispersed in poly(vinylidenfluoride) was shown to be responsive to benzene at room temperature according to the applied voltage. At high voltage the relative resistance of the film increased linearly (r = 0.92) with benzene concentrations resulting in a detection limit of 10 ppm.

In 2005, Tamaki [98] presented a review of MOx sensors in which VOCs were cited in a few articles. The lowest limit of detection (1–3 ppb for BTX) was attained using an Au-SnO_2_ MOx sensor heated at 400 °C combined with pre-concentration [99] as in a micro-GC. Without pre-concentration, a Pd-WO_3_ sensor heated at 400 °C was reported to be able to measure between 10 and 1000 ppb of aromatics [100]. Other references for MOx sensors including WO_3_-SnO_2_, CuO-SnO_2_, SmFeO_3_, Ti-W-O and Pt/Al_2_O_3_-WO_3_, reported measurement capacity over 1000 ppb that are not relevant for ambient air monitoring.

In 2009, Ke et al. [101], developed a MEMS-based benzene gas sensor consisting of a quartz substrate, a thin-film WO_3_ sensing layer, an integrated Pt micro-heater and Pt interdigitated electrodes (IDEs). The sensing process used oxidation of the heated WO_3_ sensing layer caused by benzene, which lead to changes in the electrical resistance between the IDEs. At an optimal working temperature of 300 °C, the sensor had a high degree of sensitivity, a detection limit of 0.2 ppm and a rapid response time (35 s). 

Wen et al. [74], developed a SnO_2_–TiO_2_ based sensor doped with Ag ion powder prepared using the sol–gel method. The authors showed how the sensor can exhibit remarkable selectivity to each VOC by tuning the voltage of the heater and hence the operating temperature of the sensor. Further investigations based on quantum chemistry calculations showed that the difference of orbital energy of VOC molecules might be a qualitative factor affecting the selectivity of the sensor. In this paper, the ratio of resistance (*R*_0_) in air to that in the tested gas (*R*) was found to be about 60 for a mixture of 200 ppm of ethanol. Setting that as the limit of detection would correspond to *R*/*R*_0_ ratio higher than 3 and assuming linearity between *R* and the ethanol level, the limit of detection would be about 10 ppm (equal to 200/60 × 3). However, this value is indicative since *R* and the gas pollutant show generally a logarithmic relationship that would result in a lower detection limit. However, the main interest of the author was to demonstrate the improvement in selectivity rather than the best detection limit.

In 2010, Zeng et al. [102] demonstrated and explained the mechanism behind the improvement in the sensitivity for VOCs monitoring in indoor air by adding TiO_2_ dopant to SnO_2_ sensors. The paper focuses on the explanation of the improved sensitivity rather than estimating the detection limit of the sensor. Similarly, Kadosaki et al. [103] looked at the most suitable oxide materials among WO_3_, SnO_2_, In_2_O_2_ and ZnO for total volatile organic compound (TVOC) measurements in indoor air. The authors showed that SnO_2_ and more clearly WO_3_, were the most sensitive oxide materials among those tested. Among oxy, halogenated, aromatics, aliphatic hydrocarbons, terpenes, esters and aldehydes, the halogenated and aliphatic hydrocarbons gave the lowest responses of any of the oxide materials. For the aromatic hydrocarbons, higher sensitivity (up to four times more) was found for compounds with functional groups (methyl groups) added to simple rings or double bonds both for SnO_2_ and WO_3_ sensors: higher response for terpenes, toluene, xylenes, ethylbenzene, styrene and trimethyl benzene than for benzene. However, the authors concluded that a simple SnO_2_ or WO_3_ sensor is not able to detect ppb levels of VOCs. They also investigated the addition of noble metals to improve sensitivity of SnO_2_ or WO_3_ sensors. Best results were obtained from the addition of Ag to SnO_2_ sensors for sensing heptanes, while for WO_3_ sensors Au is for toluene and heptane sensing and Pd is added for toluene and trichloroethylene sensing. The addition of noble metals had smaller effects on In_2_O_2_ and ZnO sensors than on SnO_2_ or WO_3_ sensors. The authors studied the effect of Pt and Pd optimal amount, size of dopant and temperature heater for the highest response of the SnO_2_ sensor to halogenated and aliphatic hydrocarbons. In this paper, the ratio of resistance (*R*_0_) in air to that in the tested gas (*R*) was found to be about 6 for a mixture of 1 ppm of toluene using SnO_2_ sensor operated at 250 °C with 0.5 wt % of Pt and 0.5 wt % of Pd. With a LoD set to a *R*/*R*_0_ ratio of 3 and assuming linearity between R and toluene level, the limit of detection would correspond to 0.5 ppm (equal to 1/6 × 3). However, this value is indicative since *R* and gas pollutant generally show a power law (Equation (1)) that would result in a lower detection limit. The authors concluded that for some compounds a 15-fold increase in sensitivity could be reached according to the additive composition, film size and according to the temperature of the sensor heater. Surprisingly they did not study the optimization of WO_3_ sensors that seemed to be more sensitive than SnO_2_ sensors. However, the main interest of the authors was to demonstrate the improvement in selectivity rather than the best sensitivity.

Metal oxide semiconductor gas sensors generally respond to a large variety of gases. To achieve selectivity the well-established temperature cycled operation (TCO) can be used [104]. The signal output of TCO can be seen as a virtual sensor array providing multichannel information. Data are typically processed by discrimination and quantification algorithms like e.g., linear discriminant analysis (LDA) and partial least square regression (PLS) [105]. Earlier work shows that TCO can improve the sensor response of MOS sensors to VOCs like e.g., ethanol, toluene and benzene [106,107]. In the TCO, non-equilibrium states of the sensor can be obtained, that are more sensitive than all equilibrium states at different temperatures. The response can be increased for more than three orders of magnitude for 1 ppm ethanol. However, the concentration dependency no longer fulfils Equation (1). The response at concentrations below 10 ppb decreases rapidly with an exponential response characteristic. Therefore, small gas concentrations of few ppb and below are hard to detect and quantify.

Commercial sensors using temperature cycle operation have been shown in laboratory studies to detect toxic VOC in the ppb range [11]. Some sensor manufacturers provide information on TCO parameters in their data sheet e.g., Figaro Engineering for the detection of CO (TGS-2442) but in most case specific use of TCO is implemented by sensor system manufacturers [108]. Sensor systems for VOC detection based on TCO have been reported from several companies e.g., 3S GmbH (DE) and NanoSense (FR). The TCO mode may be also applied to Silicon Carbide Feld Effect Transistors (SiC-FET) that reach sub-ppb sensitivity most likely by keeping the selectivity through the use of LDA and PLS statistical methods [109,110]. All the characteristics of the above-mentioned device are listed in Table 4 and Appendix A.

## 6. Spectroscopic Methods

In 2013, Allouch et al. [112] reviewed recent research studies for optical and colorimetric-based portable devices for high sensitive and real time BTEX analysis. Of all the instruments included in this review, only two systems were suitable for the low ppb range.

The first system used a micro-fluidic UV portable spectrometer with a silicate absorbent and later thermal desorption which resulted in a limit of detection of about 10 ppb for hourly values [113]. The system was further improved to reach a limit of detection of about 1 ppb for 30-min averages, although whilst resulting in a more complicated setup including a pulse pump system and new detection cell [114]. The second instrument [115] was based on the proportional variation of the reflected light intensity when BTEX gases are present in a detection tube which contains an optical fiber coated with a polymeric sensitive film. The device is composed of a concentration system (12 cm length) and a detection cell (7.2 cm length). BTEX were injected as liquid into the injection cell and then vaporized. Thus, the gaseous BTEX were transmitted to the glass tubing containing a poly-dimethysiloxane (PDMS) layer for the adsorption and desorption processes and finally to the detection tube that contained the coated sensitive film (thin film of polysiloxane) optical fiber. The author obtained the most convincing results with a diode wavelength of 650 nm and a sampling time of 25 min at 200 mL/min. Gas chromatography-flame ionization detector (GC-FID) has been used to control the results. The detection limit was found to be around 2.5 ppb for benzene without the need for a pre-concentration step. Despite the fast, real-time and on-site monitoring of BTEX provided by this sensor, it is still consuming chemicals. In addition, polymer-based sensing is limited in terms of durability.

Maruo et al. [116] developed a portable device for formaldehyde monitoring aiming at a simple and inexpensive sensor. It is based on specific colorimetric reaction between formaldehyde and a β-dikétone in a glass substrate where it remains stable for a long time. The sensor system includes two LED light sources and two photo-diodes to measure the absorbance of the lutidine derivative formed in the substrate at two wavelengths in order to correct the temperature/humidity effects of the absorption mechanism. The absorbance difference of the sensing element was regularly measured by the monitoring device and the result was converted into formaldehyde concentration. The detection limit was found to be 10 μg/m^3^ for a 30 min sampling. Table 5 and Appendix A list the sensitivity of the above methods.

## 7. Portable Miniaturized and Micro Gas Chromatographs

Portable gas chromatographs used for the determination of VOCs in air quality assessment can be classified into miniaturized chromatographic systems, micro gas chromatographs (μGC) or lab-on-a-chip type (LOC) and portable gas chromatograph analysers. 

Low power consumption sample processing, column programming, detection systems and data handling have been combined to reduce the size and weight of GCs for portable use. The simplest may consist of little more than an ambient temperature injector, column, and detector, while the most complex may have every feature of an advanced laboratory instrument. Portable GCs may be based on semiconductor chip processing or assembled from discrete components

### 7.1. Principle of Portable and Micro Gas Chromatographs

Micro gas chromatographs (μGC) combine micro GC columns with either PID or MOx sensors. The first miniaturized GC, created by Terry et al. at Stanford University in 1979, was fabricated on a silicon wafer using photolithography and chemical-etching techniques [118]. The μGC included an injection valve and used an internally mounted thermal conductivity detector. The dimensions of the column were 1.5 m long, 40 μm deep and 200 μm wide with a rectangular cross-section. The complexity of evenly coating a rectangular channel and in the overall miniaturization of the other components had for major consequences to weaken the resolving power of the column compared with modern standard columns. In 2013, another review [119] looked into the pre-concentration step needed to decrease the limit of detection of VOC sensors.

The ion mobility spectrometer (IMS) can be considered as a sub-class of chromatographic separators. The basic principle of IMS is a time-of-flight measurement. Gaseous molecules are ionized by a radioactive source and the resulting ions are then accelerated over a short distance and their time-of-flight can be determined. The IMS is different from the mass spectrometer in that it operates under atmospheric conditions and does not need large and expensive vacuum pumps. Because of this, IMSs can be easily miniaturized.

### 7.2. Commercially Available Sensors

A few instruments exist in this category:

- the Person-Portable GC/MS of INFICON models Explorer Portable GC, HapSite ER and HapSite smart/smart plus [120,121]
- an automated vapor sampling with transfer line between a gas chromatograph and a ion mobility spectrometry (IMS) the Environmental Vapor Monitor (EVM II) of Femtoscan [122],
- a portable gas chromatograph with 3 columns at 40–80 °C, PID sensor (10.6 eV) and internal cylinder for Ultra High Purity N2/Zero Air as carrier gas, the INFICON model Explorer Portable Gas Chromatograph [123]
- and portable GCs, the Defiant Technologies model Frog 4000 [124], the Electronic Sensor Technology zNose, model 4600 [125], the PID Analysers model 312 [126] and GC Companion [127].

These instruments reach the desired sensitivity and selectivity (Table 6 and Appendix A). However, the price of such instruments (between 15k and 100k €) limit their applicability. The Bentekk (DE) measurement device [128] is a promising portable gas chromatograph coupled with photo ionization detection. It can selectively analyse more than one VOC within 30 s. The instrument does not need an external carrier gas and it weighs about 1 kg. In the current state, the limit of detection is stated as 50 ppb in the data sheet, in a personal communication it was given as 25 ppb and the aim of the company is to reduce it to 15 ppb.

Other instruments consist of the μRAID [129] and the RAID-M100 [130], two IMS portable hand-held detectors that are used in the field of military, emergency response and security services manufactured by Bruker. They are able to measure toxic substances in the ppb to ppm range. They are not included in Table 6 because the manufacturer does not provide any information related to the monitoring of aromatics in ambient air. The main objective of this instrument seems to be military applications. In 2006, Statheropoulos et al. [131] conducted a study of acetone in expired air using the EVM II. The limit of detection of acetone with the EVM II was found to be lower than 100 ppb.

### 7.3. Literature Survey

In 2015, Marc et al. [132] described the state of the art for micro chromatographic systems (μGC) of the lab-on-a-chip type (LOC) and for portable analysers for the detection and identification of specific compounds present in ambient air (indoor or outdoor). In this review, the authors cite the lab-on-a-chip sensor developed by Halliday et al. [133]. This preliminary development consists of a planar 2-dimensional GC chip micro-fabricated system using a PID detector suitable for the separation of VOCs and compatible for use as a portable measurement device. This low-power device showed good separation performance for a small set of VOCs and promising preliminary results for the separation of ppm gas mixtures of a set of VOCs.

Sanchez et al. [134] presented a hybrid micro-system to get a sensitive and selective detection of VOC in air. Micro-fabrication technology was applied to the development of a gas chromatographic micro-column placed in an oven at 303 K, placed upstream of the gas sensor. It included a simple MOx sensor (Microsens, Neuchatel, Switzerland) whose conductance was measured at 773 K. The carrier gas consisted of dry N_2_ (80%) and O_2_ (20%). Special attention had to be paid to the pressure of the carrier gas. The sample was formed by a gas mixture of benzene, toluene and two isomers of xylene eluted in synthetic air. This prototype, beyond its small size and its great chemical discrimination, provided fast responses and offered best results with very low temperatures of the GC micro-column. No information about the limit of detection or validation is given in this paper apart from the ability to separate synthetic mixtures including BTX. Sensitivity in the sub ppm range was expected with this system.

Zampolli et al. [135,136,137] developed a μGC for the detection of sub-ppb BTEX. The systemic approach includes the study of innovative pre-concentration materials, micromachining of GC components and devices, the fabrication and characterization of complete system prototypes including a pre-concentration unit, separation unit and detection unit. The pre-concentration unit is based on a Micro-Electro-Mechanical System (MEMS) pre-concentration column packed with an appropriate chemical phase that traps aromatic compounds during sampling. When heated, it injects the pollutant into the separation unit consisting of the MEMS column filled with a commercial stationary phase. The platinum heater and temperature sensor integrated on the GC column allow temperature control and modulation. In this study, the detection unit consist of a MOx sensor [138]. The air samples and the on-board generated carrier gas flow are provided by two mini pumps and managed by two mini valves. A dedicated software is used to manage the whole system and program it for a specific measurement sequence. The prototype is currently being optimized with a PID sensor in order to make it commercially available.

In 2009, a hybrid system for rapid detection and analysis of BTEX was developed by Huang et al. [139]. The system combined a selective and sensitive sensing element with a fast and miniaturized chromatographic separation method. The sensing element consisted of an array of micro-fabricated quartz crystal tuning forks modified with selective molecularly imprinted polymers. The sensitivity and selectivity of the sensing elements together with the help of the separation provided fast detection and analysis of BTEX in real samples containing highly concentrated interfering agents without pre-concentration or heating of columns. The low cost, low power consumption and small size of the hybrid device were suitable for occupational health, industrial safety, and epidemiological applications. A linear dynamical range from about 5 ppm and up to 250 ppm was reported. This work seems to have been continued by Chen et al. [140] to build a wireless hybrid chemical sensor for detection of environmental VOCs with similar sensing principle. The device was wireless, portable, battery-powered, cell phone operated and it allowed reliable detection in ppb of BTEX in the presence of complex interferents. 

In 2012, Bae et al. [141] presented a portable gas analyser that consisted of a micro-flame ionization detector (micro-FID) and a μGC. Both components were integrated in a “lunchbox” sized housing with all the peripherals to operate the micro-GC/FID without an external power and gas supply. The total size of this μGC/FID lunch box was 24 × 20 × 10 cm^3^ with 4 kg mass. An electrolyser in the lunchbox produced pure hydrogen and oxygen for the micro-FID, eliminating the need for gas tanks on-board. The instrument was aimed at analysis in NASA planetary exploration missions, cabin air monitoring in spacecraft or in the international space station. The separation/detection capability of the NASA SMAC list of compounds including benzene, toluene, ethylbenzene, xylenes (BTEX), and other organic molecules was demonstrated. The authors concluded that the instrument would be able to detect VOCs of less than 0.1 ppm concentration levels.

The Zeller research team at the University of Michigan has designed several MEMS-μGC prototypes for continuous monitoring of VOCs [142]. The MEMS-μGC was adapted for the analysis of low- and sub-ppb concentrations of trichloroethylene (TCE) vapours in complex mixtures of indoor air contamination. The μGC prototype employed a microfabricated pre-concentration focuser, dual microfabricated separation columns, and a microsensor array. These were interfaced to a non microfabricated front-end pre-trap and high-volume sampler module to reduce analysis time and limits of detection (LOD). Selective preconcentration and focusing were coupled with rapid chromatographic separation and multisensor detection for the determination of TCE in the presence of up to 45 interferences. Constant sensitivities over the range of captured TCE masses tested (i.e., 9–390 ng) were observerd. TCE was measured in a experimental mixture at 120 ppt, with a projected LoD of 40 ppt (4.2 ng captured, 20 L sample) with 36 of maximum response time (sampling and analytical cycle). Short- and medium-term (1 month) variations in retention time, absolute responses, and response patterns were found within acceptable limits. The application of the μGC was demonstrated through laboratory testing. The prototype was then implemented in real case studies of indoor air analysis including validation against reference measurements [143,144] showing low bias (generally within ±25%), linearity of the μGC in the range between 4.8 and 77 ppb and a re-evaluated LOD of 52 ppt. A subsequent publication by Bryant-Genevier et al. [145] was aimed at improving and applying chemometric methods for use with micro-GC chemiresistor sensor arrays. This paper described the use of hybrid multivariate curve resolution method, which combines evolving factor analysis (EFA) with alternating least squares (ALS), to the analysis of partially overlapping peaks from vapours measured by a microsensor-array gas chromatograph detector. Calibrated response patterns are used as inputs for the ALS refinements of EFA-extracted responses. This provides a highly differentiation capacity sufficient enough to differentiate the composite peak components in 124 of 126 cases (98%) and to quantify them to within ±30% of actual values in 95 of 126 (75%) cases. Recently, Bryant-Genevier [146] published an article describing the development of a highly effective MEMS pre-concentrator focuser to be associated with the μ-GC developed before.

Nasreddine et al. [147] reported on a miniaturized GC/PID system dedicated to BTEX monitoring in near to real time conditions at ppb level for indoor. The system consists of a six-port valve, a capillary column and a mini-photoionization detector operating at very low flow rate (lower than 4 mL/min). The total analysis time for a single run was set to a maximum of 10 min. A detection limit of 1 ppb was found for benzene and toluene with hydrogen and nitrogen as carrier gases and less than 3 ppb for other compounds of the family.

## 8. Electronic Noses and Sensors Arrays

### 8.1. Principle of Operation

Another class of measurement devices consists of e-noses and sensor arrays, which are devices that contain several simple sensors of different type. They use mathematical pattern recognition algorithms in order to compare and recognize gaseous samples. Among the different algorithm used to make the sensor array more specific than each single sensor, smart pattern recognition software are commonly based on neural networks. Sensor arrays are often part of devices called “electronic noses” or “e-noses”. This name is derived from the fact that (part of) their job is to detect odours. Each element in the sensor array responds to a number of different chemicals or classes of chemicals. The individual selectivity of each element is not required as the array of sensors should contain as much chemical diversity as possible. This diversity gives to the e-nose the ability to respond to the largest possible range of analytes. Using a fingerprint method over the collection of sensors, the e-nose is able to classify and identify analyte. E-nose systems are not expected to be readily applicable for accurate quantitative benzene measurement since many of them have another main target application and they are costly. It is reported that cost for an e-nose system ranges from US $20,000 to $100,000 in Europe, the United States, and Japan [148].

### 8.2. Commercially Available Electronic Noses and Sensor Arrays

The μChemLab developed by Sandia National Laboratories does not seems to have application in the field of BTEX and VOC monitoring in ambient air [13]. It is however, an example of a hand-held miniaturized GC instrument for VOC measurement that incorporates a microfabricated etched column. It comprised a pre-concentrator consisting of a thin silicon nitride membrane supporting a patterned metal film-heating element, a 1 m long 100 × 400 μm high-aspect ratio GC column on a silicon wafer and an array of surface acoustic wave sensors.

Some examples of commercially available sensor arrays are the eNose of Comon-Invent consisting of 4 different semi-conductor sensors, the Aerekaprobe of The eNose Company using a system with 1 to 12 micro-hotplate temperature modulated metal-oxide sensors, and the Airsense PEN3 consisting of 10 different metal oxide single thick film sensors working at temperatures from 350 to 500 °C. The range of measurable gas concentrations of these sensor arrays varies from ppb to upper ppm.

### 8.3. Literature Survey

A few articles already propose reviews of electronic nose technologies. In particular, Röck et al. [149] and Wilson and Baietto [150] published reviews papers in which they classify, describe and give examples of commercially available e-nose and their possible use. They are particularly interesting for odour detection and monitoring.

Lee et al., 2002 [151] presented an array of ten different gas MOs-sensors (SnO_2_) integrated on a substrate to identify various kinds and quantities of VOCs, such as benzene, toluene, ethyl alcohol, methyl alcohol, and acetone. The sensor was fabricated using silk printing methods on an alumina substrate. The study showed a high and broad sensitivity and reproducibility to low concentrations based of the nano-sized sensing materials. The author also implemented the sensing signals of the array in an artificial neural network with an error-back-propagation learning algorithm. Both simulation and experimental results showed that this device using neural network was able to recognize and quantify various kinds of VOCs.

Srivastava [152] showed the possibility of using an array of SnO_2_ doped gas sensors (Pt, Pd and Au) for the detection of VOCs. Using a three-layer feedforward neural network classifier with improved accuracy based on mean and variance of the individual gas-sensor combination, the author showed that the e-nose was able to successfully identify seven VOCs even with noisy data. However, there was no further attempt to quantify the different VOCs.

Penza et al. [153] reported that sensors coated with nanofilms and nanocomposites can show improved sensitivity for organic gases. They developed a multi-sensor device using Langmuir-Blodgett deposited multilayers of single-walled carbon nanotubes on surface acoustic waves, quartz crystal microbalance, and standard silica optical fibre. This multi-sensor was simultaneously exposed to six VOC vapours. The authors reported a good correlation between the sensing mechanisms and refractive index of the sensors. Based on the experiment results, the potential use of multi-sensors based on carbon nanotubes and pattern recognition techniques was suggested for the efficient analysis of VOCs.

Han et al. [154] proposed to use an array of nanosized sensors to detect a mixture of organic gases. The nanostructured array was based on thin film assemblies of alkanethiolate-monolayer-capped gold nanoparticles (2 nm) formed by a molecularly mediated assembly using mediators or linkers of different chain lengths and functional groups. Each array was tested for benzene, toluene, xylenes, nitrobenzene, 2-nitrotoluene and 3-nitrotoluene measurements and showed linear responses. The response was then analysed by artificial neural networks with principle component analysis technique. However, in this study, the tested concentration of organic compounds were between several ppm to thousands of ppm, far from the targeted ambient air application.

In 2006, Wolfrum et al. [155] presented a sensors arrays based on 14 MOx Figaro TGS 2602 sensors with different power supplies in the range 1 to 5 V plus a temperature and a humidity sensor. After laboratory calibration, the Partial Least Squares technique was used for data treatment and quantification of up to 10 VOCs in the range 10 to 300 ppb adapted for indoor air monitoring.

In 2008, De Vito et al. [8] presented an e-nose made of seven low-cost metal oxide sensors manufactured by Pirelli Labs (IT). In this paper, the authors presented a field calibration method able to solve the lack of selectivity and stability of MOx using a neural calibration for the prediction of benzene concentrations using a gas multi-sensor device (solid-state) designed to monitor urban environment pollution. The author presents a feasibility study of a sensor fusion algorithm using conventional air pollution monitoring station as reference data. The results were assessed by means of prediction error characterization throughout a 13-month campaign. The authors showed that a neural calibration using only a small number of measurement days was able to limit the absolute prediction error for more than six months.

## 9. Validation Studies

A small number of VOC sensor performance evaluations can be found in the literature. An evaluation of the ppbRAE3000 is reported in the ISO/FDIS 16000-29:2014, Indoor Air–part. 29: test methods for VOC detectors [156]. The instrument was tested in an exposure chamber at 24 °C and 50% of relative humidity. VOC mixtures were prepared by dilution of gas cylinders with zero air using Mass Flow Controllers delivering a total flow of 800 mL/min. Unfortunately, the VOC level of concentration were very high, generally several ppm (toluene equivalent between 0.1 and 6 mg/m^3^), which correspond to the expected VOC levels in indoor air studies. Reference values for the VOC mixtures were established by GC-MS. The responses of the ppbRAE3000, were given as to isobutylene equivalent without using the correction factor of each compound in the VOC mixtures [157]. The difference of indications between the ppbRAE3000 and the GC-MS measurements for each VOC mixed gas (toluene, a mixture of two VOC and mixture of six VOC) were 27.9%, −31.6%, and 4.9%, respectively. These tests are not adapted to the field of air quality monitoring both because of the level of concentration being tested and the lack of identification of the performance for each compound.

The United States Environmental Protection Agency (US-EPA) has published a report about the evaluation of VOC sensors [76]. This report summarizes the results of evaluations performed on low cost (<$2500) sensors. A total of five devices were tested under laboratory and field evaluation conditions with one of those being an EPA developed device which used a commercially-available VOC photoionization detector (PID) as the sensing element. The laboratory evaluations involved testing the devices to a stepwise pattern of VOC concentrations at ambient levels (benzene < 25 ppb) in an exposure chamber. The selected sensors consisted of the UniTec SENS-IT, AirBase CanarIT, CairPol CairClip, APPCD PID (PID sensor developed by US-EPA) and ToxiRAE Pro. It was shown during the lab experiments that the APPCD-PID and UniTec SENS-IT responses appeared well correlated with reference values of benzene and benzene/1,3-butadiene/tetrachloroethylene mixtures at concentrations well below 25 ppb for benzene and 75 ppb for the VOC mixtures. These results demonstrate the detection capabilities of the two sensors at these levels that would be useful for non-industrial environmental monitoring. However, issues with noisy response outputs were observed that limited the usability of some of the named devices. The author emphasized the need of improving some key features as ease of use, simplicity of operation, and ease of data recovery. They concluded that low cost VOC technologies appeared to be limited in both their capability and variety of technologies being employed. PID components still appeared to be the prominent sensing element available. However, these came with inherent pros and cons (selectivity, drift over time, …) which must be considered when trying to use such a device to estimate VOC concentrations under a variety of monitoring scenarios.

The devices were first evaluated for their response to a single VOC (benzene). If the device revealed some ability to detect benzene at even 25 ppb it was then challenged with an atmosphere consisting of three VOCs (benzene, 1,3-butadiene, and tetrachloroethylene). These compounds were selected because of the availability of well-qualified test gases and the fact they represented a variety of VOC moieties (structural variability). Next Generation Air Monitoring (NGAM) devices were deployed at an outdoor near road test platform for an extended period where wide variability of VOC conditions were expected to exist. The research plan involved direct comparison of the NGAM response to GC reference data from collocated measurements obtained at the test site. Reference data were ultimately not available for the intended comparisons (instrument malfunction and insufficient resources to conduct a timely repair). Therefore, field data provided were limited to non-reference comparisons between NGAM devices. Such comparisons, providing a non-quantitative assessment of true VOC response still have the potential of yielding useful information on the relative response characteristics of the NGAM VOC devices evaluated.

## 10. Conclusions

In this paper, a literature review of portable and low-costs sensors, sensor devices and miniaturized measurement devices for the monitoring of benzene in ambient air has been presented. Based on information provided be the manufacturers (datasheet) and the existing literature, we were able to draw a general overview of the technology currently in development or commercially available with a figure in Appendix A giving a graphical overview of the LoD of all sensor systems.

In general, the main drawback of these devices are the lack of sensitivity and/or selectivity to benzene. Most of the MOx and amperometric OEM sensors are not able to reach levels lower than 100 ppb of benzene, although a few embedded sensors device show a sensitivity to few tens of ppb. Their limit of detection are two to three orders of magnitude too high for monitoring benzene in ambient air at the desired 1 ppb limit of detection. On the other hand, PID sensors are intrinsically not selective enough. Only devices including a filtering or absorbing cartridge (PID-based instruments) may be an alternative at levels from 10 ppb for the ones selective to benzene (Tiger Select Benzene and UltraRAE3000). However, their limit of detection is still about one order of magnitude too high. In addition, multi-sensors coupled with artificial neural network algorithms can be considered a feasible candidate sensor system

Portable gas chromatographs and ion-mobility spectrometers would be both sensitive and selective. Unfortunately, they are simply too expensive for the low-cost sensor market. Among research studies, we could find prototypes or semi-commercial systems including SiC-FET and multi MOx sensors operated in Temperature Operation Cycle that offer both ppb or sub ppb sensitivity with selectivity to specified VOCs. A few advanced prototypes of miniaturized Gas Chromatographs can be found in literature with equivalent or better sensitivity and selectivity.

Based on the above review, a list of potential sensors/sensor based devices which show the most interesting performances can be set. Among the commercial OEMs sensors, only the VM semiconductor sensor from Aeroqual with its 0.001 ppm of limit of detection, and both PID sensors from Mocon Baseline (model Blue, item 045-014) and Alphasense (model PID-AH) with respectively 0.00025 and 0.0005 ppm of limit of detection show a real interest for benzene measurement in ambient air monitoring. 

Portable devices based on PID sensors, such as the UltraRAE 3000 and ppbRAE 3000 from RAE (LoD = 0.05 and 0.001 respectively), the Tiger Select from IonScience (LoD = 0.01 ppm, with a resolution of 0.001 ppm) and the Multi-PID 2 from Draeger (LoD = 0.05 ppm) generally show a good selectivity using selective filtration or miniaturized GC technology. However their sensitivity is consequently lower. Even though they are too expensive, the X-PID from Bentekk (LoD = 0.05 ppm, ongoing developpment to achieve 0.015 ppm) and the Frog 4000 from Defiant (LoD in the ppb range) were included based on their performances (usually both sensitive and selective) and also their small dimensions.

Among research studies, one can find prototypes or commercial systems comprising portable UV spectrometers, and multi MOx sensors [9] or SiC-FET [110] operated in Temperature Operation Cycle that offer both ppb or sub ppb sensitivity with selectivity to specified VOCs. A few advanced prototypes of miniaturized gas chromatographs can be found in literature with equivalent or better sensitivity and selectivity. Finally, multi-sensors coupled with artificial neural network algorithms can be also considered a possible candidate sensor system.

## Figures and Tables

**Table 1:** Sensitivity of PID sensors for the measurement of benzene or other VOCs.

	Model	Manufacturer	LoD for Benzene, ppm	Sensitivity for IBE, V/ppm	Selectivity, Known Interferents	Stability, Drift	Range for IBE, ppm	t_90_, s
[15]	ppb MiniPiD white	Ion Science	0.5	0.025	VOCs with IP lower than the lamp output are detected. Interference from humidity and temperature	calibration periodicity < 1 month	0.001–40	2
[16]	piD-TECH eVx Blue 045-014	Baseline-Mocon	0.25	1.125			0.0005–2	a few seconds
[17]	piD-TECH plus, 043-235	Baseline-Mocon	2.5	0.125			0–20	<5
[18]	PID-AH for VOCs	Alphasense LTD	0.5	>0.020			0.0005–50	<3
[19]	Multi-PID 2	Dräger	0.050	Not relevant	Benzene absorption tube	No data	0.100–2000	3
[20]	Model 102+	PID Analysers	0.001	Not relevant	All VOCs with IP < 9.6, 10.2 or 11.7 eV	No data	0.001–20	1
[21]	VOC-Traq	Baseline-Mocon	0.005	Not relevant	VOCs with IP < 10.6 eV	No data	0.010–20	10
[22]	Club	Ion Science	0.0005	Not relevant	All VOCs with IP < 10.0 eV	No data	0.001–5000	13
[23]	Tiger Select benzene	Ion Science	0.010 (resolution 0.001)	Not relevant	Use benzene pre-filter	No data	0.001–5000	<2
[24]	AdvancedSense DirectSens IAQ	Graywolf	0.0025	Not relevant	VOCs with IP < 10.6 eV	No data	0.005–20	<60
[25]	UltraRAE 3000	RAE Systems	50	Not relevant	9.8 eV lamp and benzene tube	No data	0.050–200 (benzene)	60
[26]	ppbRAE 3000	RAE Systems	Resolution 1 with 10.6 eV lamp		VOCs with IP < 10.6/9.8 eV	No data	0.001–10	2

IBE: isobutylene, IP: ionization potential [27], t_90_: time response, time for reaching 90% of final value.

**Table 2:** Sensitivity response time and limit of detection of the commercially available amperometric sensors.

	Model	Supplier	LoD, ppm	Sensitivity, μA/ppm	Selectivity, Known Interferents	Stability, Drift in ppm	Range, ppm	t_90_, s
[43]	3ETO CiTiceL	City Technology	0.1 (Resolution)	2.75 ± 0.5	CO, HC, and VOCs	Zero: 2, Baseline: 0–1, Span: <5%/year	0–20	<140
[44]	4ETO CiTiceL	City Technology	0.1 (Resolution)	1.9 ± 0.5	VOCs in general	Zero: 4, Baseline: 0–3, Span: <5%/year	0–20	<120
[45]	7ETO CiTiceL	City Technology	0.1 (Resolution)	2.25 ± 0.65	Ethanol ≈ 55%; MEK ≈ 10%; Toluene ≈ 20%; CO ≈ 40%	Zero: 2, Baseline: 0–1, Span: <5%/year	0–20	<140
[46]	ETO-A1	Alphasense LTD	0.1	2.0 to 3.2	The bias voltage is set for ETO and needs adjusting for other VOCs	No data	0–100	<150
[47]	ETO-B1	Alphasense LTD	0.1	2.0 to 3.2		No data	0–100	<150
[48]	ETO/M-10	MembraporAG	0.05 (Resolution)	2.0 ± 0.5		Zero: no data, Baseline: 0–1, Span: <2%/month	0–10	<140
[49]	ETO/C-20	Membrapor AG	0.1 (Resolution)	2.5 ± 0.6	Interference evaluated for a list of VOCs	Zero: no data, Baseline: 0–1, Span: <2%/month	0–20	<140
[50]	EC4-10-ETO	SGX Sensortech	0.1 (Resolution)	1.9 ± 0.8	CO, HC, and VOCs	Zero: −0.2–2.5 μA, Baseline: 0–2, Span: <2%/month	0–10	<120

ETO: ethylene oxides, C2H5OH: ethanol, MEK: methyl ethyl ketone (CH_3_C(O)CH_2_CH_3_).

**Table 3:** Sensitivity response time and limit of detection of the commercially available MOx sensors.

	Models	Manufacturers	LoD, ppm	Sensitivity log(Rs/R_0_)/log(ppm)	Selectivity, Known Interferents	Stability, Drift	Range, ppm	t_90_, s
[78]	VM	Aeroqual	Res.: 0.001	No data	negative response with oxidising gases, positive response with combustible gases	No data	0–25	60
[79]	iAQ-100	AppliedSensor	VOC + CO_2_: 350	No data	alcohols, aldehydes, aliphatic hydrocarbons, amines, aromatic HC, CO, CH_4_, LPG, Ketones, Organic acids	No data	VOC + CO_2_: 350–2000	15 min
[80]	iAQ-2000	„	No data	No data		No data	CO_2_: 450–2000	15 min
[81]	iAQ-engine	„	CO_2_: 450	No data		No data	CO_2_: 450–2000	15 min
[82]	AS-MLV	„	About 1	No data		No data	CO_2_: 450–2000	seconds
[83]	TGS 2201	FIGARO USA	i-butane: <1	i-butane: −0.26	CO, H_2_, CH_3_OH, other HC, with similar sensitivity, NO_2_, SO_2_ and H_2_S according to load resistance	No data	i-butane: 2–100	No data
[84]	TGS 2600	„	i-butane: <1	i-butane: −0.24	CH_4_, CO, i-butane, Et-OH, (CH_3_)_2_CO, H_2_	No data	i-butane: 1–100	No data
[85]	TGS 2602	„	toluene: <1	Toluene: −0.6		No data	toluene: 1–30	No data
[85]	TGS 8100	„	toluene: <1	i-butane: −0.14		No data	i-butane: 1–30	No data
[86]	TGS 822	„	benzene: <50	Benzene: −0.67	CH_4_, CO, i-butane, n-hexane, ethanol, acetone	No data	benzene: 50–5000	No data
[87]	SP3_AQ2	FIS	<1	CO: −0.4	VOCs	No data	EtOH: 0.1 to 100	No data
[88,89]	MICS-5121/5521	SGX Sensortech	No data	CO: −0.59	reducing gases such as CO, HC and VOCs	No data	CO: 1–1000	No data
[90]	MICS-VZ-87	SGX Sensortech	No data	CO: −0.59	VOCs and CO_2_	No data	CO_2_: 400–1000	15 min
[91]	GGS 1330T	UST Umwelt-sensortechnik	No data	CH_4_: −0.186	H_2_ and CO	No data	CH_4_: −0–1000	No data
[92]	GGS 2330T	„	No data	CO: −0.361	CH_4_ hydrogen and alcohol	No data	CO: 0–1000	No data
[93]	GGS 3330T	„	No data	CH_4_: −0.227	C1-C8 hydrocarbon, CO and H_2_	No data	CH_4_: −0–1000	No data
[94]	GGS 8330T	UST Umwelt-sensortechnik	No data	EtOH: −0.227	CH_4_, CO and H_2_	No data	EtOH: 0–1000	No data
[95]	VOC Sensor (P/n 731)	Synkera Technologies	No data	EtOH: −1.166	Isobutylene (200%), CO (30%), H_2_ (10%), CH_4_ (2%), NO_2_ Negative Resp. CH_2_O (0%)	No data	EtOH: 75–700	<60
[96]	SENS 3000, SENS-IT, ETL2000	Unitec Srl	Resolution: 0.1 μg/m^3^!	No data	No data	<2.5%/6 months	benzene: 0–0.030	<3

CO: carbon monoxide, CH_2_O: formaldehyde, CH_4_: methane, CO_2_: carbon dioxide, EtOH: ethanol, HC: hydrocarbons, H_2_: hydrogen, LPG: liquefied petroleum gas, VOC: volatile organic compounds.

**Table 4:** Research study of MOx sensors for the measurement of benzene and other VOCs.

	Target Gas	Sensitive Layer	LoD, ppm	Response Time	Year
[99]	BTX	Au-SnO_2_ MOx + pre-concentrator	1–3	-	2001
[97]	Benzene	TiO_2_	10	35 s	2002
[111]	Aromatics	Pd-WO_3_ at 400 °C	Toluene: 0.010–1.000		2004
[101]	Benzene	WO_3_	0.2	20 s	2009
[74]	methanol, ethanol, acetone and formaldehyde (VOCs)	SnO_2_–TiO_2_ doped with Ag	For ethanol about 10	20 s	2010
[102]	methanol, ethanol, formaldehyde, and acetone (VOCs)	SnO_2_ doped with TiO_2_	A few 10 s	10–20 s	2010
[103]	TVOC	SnO_2_ doped with TiO_2_	toluene: 0.5	No data	2010

BTX: benzene, toluene and xylene.

**Table 5:** Sensitivity of spectroscopic method for the measurement of benzene or other VOCs.

	Target Gas	Principle	LoD, ppb	Response Time, min	Year
[113]	Benzene	Absorption/desorption/UV detection	10	60	2006
[114,117]	Benzene, Toluene, Xylene	Absorption/desorption/UV detection	1 and 0.3	30	2006, 2012
[115]	Benzene	Reflexion light	2.5	30	2009

**Table 6:** Sensitivity response time and limit of detection of the commercially available portable gas chromatographic instruments.

	Model	Manufacturer	LoD, ppm	Selectivity, Known Interferents	Stability, Drift	Range, ppm	t_90_, s
[126]	Model 312	PID Analysers	0.0005 for benzene	According to the selectivity of the GC columns. Generally this is not a problem for BETX and other VOCs	<1% over 24 h	0.0005–10000	1
[124]	Frog 4000	Defiant Technologies	A few ppb		No data	ppb range	300
[125]	zNose, model 4600	Electronic Sensor Technology	A few ppb		No data	ppb range	30
[128]	Gas Chromatography in your hand	Bentekk	0.025, on-going improvement to 0.015		No data	No data	30
[123]	Explorer Portable GC,	INFICON	0.005		No data	0.005–9999	No data
[120]	HapSite ER	INFICON	ppt depending on configuration		No data	ppt to ppm	600
[121]	HapSite smart/smart plus	INFICON	high ppt depending on configuration		No data	high ppt to ppm	900
[122]	model EVM II	FemtoScan	0.539	Separation by GC of alkanes, cyclo-alkanes, alkenes, alcohols, aromatics, ketones, esters	No data	No data	<30
[127]	GC Companion	Epananalyse	0.001–0.002 of benzene	Separation by GC	No data	No data	No data

**Figure A1:**
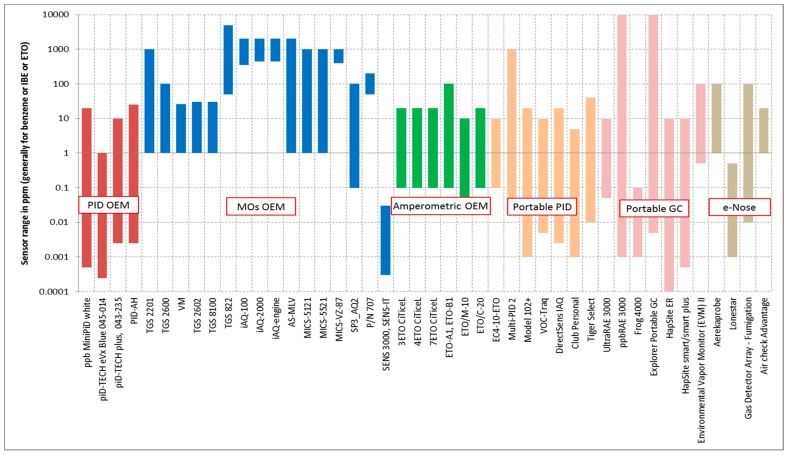
Summary table of sensors and devices for VOC measurement presented in this paper.
